# Proboscis Conditioning Experiments with Honeybees, *Apis Mellifera Caucasica,* with Butyric Acid and DEET Mixture as Conditioned and Unconditioned Stimuli

**DOI:** 10.1673/031.010.12201

**Published:** 2010-07-30

**Authors:** Charles I. Abramson, Tugrul Giray, T. Andrew Mixson, Sondra L. Nolf, Harrington Wells, Aykut Kence, Meral Kence

**Affiliations:** ^1^ Laboratory of Behavioral Biology and Comparative Psychology, Oklahoma State University, Stillwater, Oklahoma, USA; ^2^ Department of Biology, University of Puerto Rico, San Juan, Puerto Rico; ^3^ Department of Biology, Middle East Technical University, Ankara, Turkey; ^4^ Department of Biology, University of Tulsa, Tulsa, Oklahoma, USA

**Keywords:** learning, classical conditioning, insect control

## Abstract

Three experiments are described investigating whether olfactory repellents DEET and butyric acid can support the classical conditioning of proboscis extension in the honeybee, *Apis mellifera caucasica* (Hymenoptera: Apidae). In the first experiment DEET and butyric acid readily led to standard acquisition and extinction effects, which are comparable to the use of cinnamon as a conditioned stimulus. These results demonstrate that the odor of DEET or butyric acid is not intrinsically repellent to honey bees. In a second experiment, with DEET and butyric acid mixed with sucrose as an unconditioned stimulus, proboscis conditioning was not established. After several trials, few animals responded to the unconditioned stimulus. These results demonstrate that these chemicals are gustatory repellents when in direct contact. In the last experiment a conditioned suppression paradigm was used. Exposing animals to butyric acid or DEET when the proboscis was extended by direct sucrose stimulation or by learning revealed that retraction of the proboscis was similar to another novel odor, lavender, and in all cases greatest when the animal was not permitted to feed. These results again demonstrate that DEET or butyric acid are not olfactory repellents, and in addition, conditioned suppression is influenced by feeding state of the bee.

## Introduction

Considerable effort has been directed at finding olfactory and gustatory insect repellents (Isman 2006). However, even DEET (N,N-diethyl-3-methylbenzamide) one of the most widely used topical insect repellents, has alternately been shown to be an olfactory repellent (i.e. mosquitoes avoid food with DEET, Syed and Leal 2008) or not to be a repellent and only mask the odor of the potential feeding target (i.e. flies are not as attracted to odors in presence of DEET, Ditzen et al. 2008). Although finding chemicals similar to DEET in their mode of action and molecular targets would be useful, a behavioral mechanism for identifying repellents is needed. Proboscis conditioning in honeybees may provide the mechanistic model for identifying such repellents.

In the case of the honeybee, putative olfactory and gustatory repellents are used and investigated for several reasons, including public safety issues (Abramson et al. 1997), reducing the effects of harmful agrochemicals (Atkins Jr. et al. 1975a, 1975b), separating bees from honey for apicultural purposes (Graham 1992), and studying comparative aspects of behavior across taxa (Abramson 1994). Several studies in the literature suggest, for example, that *N*-octyl-, benzyl acetate, isopentil-acetate, and 2-heptanone are olfactory repellents for honeybees (Blum et al. 1978; Free 1987; Free et al. 1989). The majority of repellent studies base their conclusions on field tests (Schreck 1977).

It is generally agreed upon that the honeybee is a good learner (for reviews see Fahrbach and Robinson 1995; Menzel and Giurfa 2001; Giurfa 2007) and it is possible that the temporary decrease in honeybees observed in field tests may simply be the result of a stimulus novelty effect seen in learning paradigms (Heffernan et al. 2007). It is well known anecdotally, for those who train free-flying honeybees in conditioning experiments, that simply moving a target a few centimeters or adding a new target can easily confuse bees (see Zhang et al. 2005 for the importance of stimulus order in honeybee memory formation). It may be such confusion that gives the appearance of an olfactory repellent effect.

If a stimulus is indeed an olfactory repellent not only should it repel honeybees in field tests but, in our view, it should also be ineffective as a conditioned stimulus signaling a feeding opportunity. We also believe that an application to the antenna of the odor of a putative olfactory repellent to an already extended proboscis should produce a retraction of the proboscis as an avoidance reaction. Behavioral suppression to stimuli paired with aversive events is known in the psychological literature as conditioned suppression (Estes and Skinner 1941).

The proboscis conditioning strategy advocated here was recently used in a study investigating the repellent action of citronella to Africanized honeybees in Brazil. A field test suggested that applying citronella to cloth suspended above a feeding station reduced the number of bees visiting that station (Malerbo-Souza and Nogueira-Couto 2004). However, when conditioning procedures were employed to confirm the repellency of citronella it was found that Africanized bees readily associated the odor of citronella with feeding and that the application of citronella did not disrupt feeding (Abramson et al. 2006b). These results suggested that the strongest evidence for testing olfactory repellency in the honeybee, and probably in other insects, is the use of both field tests and conditioning protocols.

The rationale for the present experiment is to test the use of conditioning protocols on a chemical that is known to be an olfactory repellent to honeybees. Perhaps the best “known” honeybee repellent is butyric acid. Butyric acid or butyric anhydride, which quickly turns into butyric acid after application to a fume board, is used to separate bees and honey by making bees move away from honey combs (Graham 1992; Isaac and Hoffman 2002). This effect of butyric acid may simply represent a deleterious effect of the high concentration of butyric acid vapors that the bees are exposed to in an enclosed space. Alternately, butyric acid may be a true olfactory repellent and not support any conditioning. Butyric acid may support conditioning however; the taste could result in suppression of feeding if the effects were due to direct contact in the high vapor concentrations probably achieved in the closed space of a bee hive. In addition to butyric acid, a commercial formulation of the popular insect repellent DEET was also tested using classical conditioning and conditioned suppression in harnessed honeybees.

## Materials and Methods

### Subjects

The subjects for these experiments were *Apis mellifera caucasica* (Hymenoptera: Apidae) from the northeast mountainous regions of Turkey. Experiments were performed at the Middle Eastern Technical University (Orta Doğu Teknik Üniversitesi), Ankara, Turkey. The laboratory in which these experiments were conducted maintains pure lines of *A.m.* *caucasica* (Kandemir et al. 2000; Bodur et al. 2007).

All experiments were conducted during June and July of 2007. To control for calendar variables and fluctuating hive conditions, animals from all experiments were run simultaneously and selected from multiple laboratory hives contained within the apiary.

### Apparatus and Stimuli

Three conditioned stimuli (CS) were used: butyric acid (product number 100354, Teknik Kimya, Bursa), DEET (25%) (Off! Deep Woods® Insect Repellent Pump Spray, SC Johnson, Racine, WI), and cinnamon oil (Gilbertie's, Easton, CT, U.S.A.). Off! Deep Woods® Insect Repellent Pump Spray contains 25% DEET and 75% of unspecified “other ingredients.” Our rationale for using this particular formulation of DEET was that experiments have shown it to be the most effective repellent of the commercially available products containing DEET (Masetti and Maini 2006). In experiment 3, lavender oil was used as a control for the effect of novelty (Gilbertie's, Easton, CT, U.S.A.). The unconditioned stimulus (US) was a 1.80 M sucrose solution. In experiment 2, the US was either a 1.80 M sucrose solution, 0.65 M DEET, 0.90 M sucrose mixture or a 5.45 M butyric acid, 0.90 M sucrose mixture. The sucrose US was administered by dipping the tip of a 5 mm × 3 mm filter paper strip (Whatman no. 4) into the solution and applying the paper first to the antennae, and then to the now extended proboscis. When the US contained a sucrose/DEET mixture, or sucrose/butyric acid mixture it was administered with a microsyringe, first to the antenna, and then to the extended proboscis.

Approximately 3 µl of the CS chemical was applied each day to a new 1-cm2 piece of filter paper (Whatman no. 4) attached to a 20 ml plastic syringe to create a CS odor cartridge. All chemicals used as a CS were applied to the filter paper undiluted. To apply the CS, the plunger of the syringe was pulled back to the 20 ml mark and depressed. Prior research designed to directly compare automated and unautomated proboscis conditioning techniques revealed no differences in conditioning (Abramson and Boyd 2001).

### Procedure

For the proboscis conditioning experiments, foraging honeybees were captured in glass vials from laboratory hives, placed in an ice water bath, and while inactive harnessed in metal tubes. Once active, they were fed 1.80 M sucrose solution until satiated and set aside for use approximately 24 h later. Only those animals that vigorously extended their proboscis to sucrose stimulation during a pretest were used in experiments.

All proboscis-conditioning experiments used a CS duration of 3 sec and a US duration of approximately 2 sec. A conditioning trial began by placing a bee in a fume hood, after which the appropriate stimuli were introduced. After application of the stimuli, the animal was returned to a holding area and a second animal was run. A trace conditioning procedure was used where the CS was presented first followed by the US. The CS and US presentations did not overlap. If the animal extended its proboscis during the CS but before the US a ‘1’ was recorded. If the proboscis did not extend to the CS ‘0’ was recorded. Responses were recorded from visual observations. To control for possible experimenter bias, all experiments were run by a single experimenter with extensive experience performing such experiments (Abramson). Timing the sequences of stimuli was based on readings from a stopwatch. The air pressure of the depressed syringe was approximately 0.05 psi.

It is important to note that the use of the conditioned stimuli reported here is not to investigate their quantitative properties. Both cinnamon and lavender have been used in some of our previous experiments and are excellent conditioned stimuli (Abramson et al. 1997, 2001, 2006a, b, 2010). DEET and butyric acid were used to provide qualitative data on the effectiveness of these odors as conditioned stimuli, not to provide a quantitative analysis of their individual components. The DEET used was a mixture, but the content was not defined on the label.

### Experiment 1: Simple Pavlovian conditioning using butyric acid, DEET, and cinnamon as conditioned stimuli

The question of interest was whether the odor of butyric acid or DEET can serve as a cue for the onset of a sucrose feeding. One hundred and twenty bees were divided randomly into three subgroups differentiated by the type of CS (N = 40). The three subgroups were further subdivided into those that received paired CS-US presentations and those that received unpaired CS/US presentations (N = 20). Bees were randomly chosen with respect to treatment received.

Honeybees in the three paired treatments groups received 12 acquisition trials followed by 12 extinction trials in which the US was omitted. The intertrial interval was 10 min. Extinction trials were included to determine whether the effects of butyric acid or DEET could be detected by their persistent response in the absence of the US.

Honeybees in the three unpaired groups (N = 20) received 12 CS presentations and 12 US presentations in a pseudorandom order. Stimulus presentations consisted of three successive sequences of CS US US CS US CS CS US. The interval between stimulus presentations for unpaired treatment bees was 5 mins, which was half the time used for the paired treatment. A 5 min intertrial interval for unpaired treatments was used in order to maintain a 10 min interval between CS presentations. If a 10 min intertrial interval was used, the time between CS presentations would be 20 min, and any difference between paired and unpaired animals learning rates could be due to nonassociative effects of the time spent harnessed. Following the 12 CS and 12 US presentations, the unpaired experiment was terminated (no extinction trials).

An unpaired control group was included in this experiment and in experiment 2 to ensure that any conditioning observed in the paired group was due solely to the association between the CS and US. Without an unpaired control group it would be impossible to unequivocally conclude that the performance of paired animals was the result of learning as opposed to some non-associative process such as pseudo-conditioning. Moreover, we believe that including an unpaired control group is critical when mixtures such as DEET are used because it contains chemicals that are designed to make it more attractive to humans, and such chemicals may unconditionally elicit proboscis extension and/or excite the honeybee independent of its association with feeding.

### Experiment 2: Diluted butyric acid and diluted DEET as unconditioned stimuli

The question of interest was whether butyric acid and DEET mixtures diluted with sucrose could serve as unconditioned stimuli.

The design of the experiment was identical with experiment 1 with the exception that the odor of cinnamon served as the CS and the US consisted of 1.80 M sucrose solution, 0.65 M DEET, 0.90 M sucrose mixture or 5.45 M, 0.90 M sucrose/butyric acid mixture. In the US mixtures with DEET and butyric acid, mixing resulted in reduced sucrose concentration; however, in preliminary trials 0.90 M sucrose did not differ from 1.80 M sucrose in eliciting a proboscis extension response in bees kept overnight without feeding prior to testing (results not shown). This is in agreement with the empirical study of sucrose response threshold in low and high responding genetic group of bees where beyond 0.3 M of sucrose all tested genetic groups were calculated to reach saturation and respond maximally (Page et al. 1998). One hundred and twenty bees were divided randomly into three subgroups differentiated by the type of US (N = 40). The three subgroups were further subdivided into those that received paired CS-US presentations (N = 20) and those that received unpaired CS/US presentations (N = 20). Bees were randomly chosen with respect to treatment received.

As in the previous experiments, there were 12 acquisition trials followed by 12 extinction trials. The CS and US durations and the intertrial intervals were identical to the previous experiment, as was the use of a trace conditioning procedure. Honeybees in the unpaired groups were treated as in the previous experiment.

### Experiment 3: Suppression of proboscis extension

To determine whether the odors of butyric acid and DEET would suppress an extended proboscis when the proboscis was extended by learning or reflex stimulation, a variation of the conditioned suppression technique originally developed by Estes and Skinner (1941) was utilized. Previous research we have conducted over a number of years has repeatedly shown that honeybees readily learn to retract their proboscis while drinking high molarity sucrose solutions in response to stimuli predicting electric shock (Abramson 1986; Abramson and Bitterman 1986a, 1986b; Smith et al. 1991).

As in experiment 1, approximately 3 µl of butyric acid or DEET was applied each day to a new 1-cm2 piece of filter paper (Whatman no. 4) attached to a 20 ml plastic syringe to create a CS odor cartridge. In addition, CS odor cartridges were prepared for the cinnamon and lavender odors. All CS chemicals were applied to the filter paper undiluted.

A unique aspect of the study was that we elicited proboscis extension either by a conditioned stimulus that was previously paired with a sucrose US (i.e. learned extension) or directly by stimulating the antennae with sucrose (i.e. reflexive extension). Such a manipulation is new in the olfactory repellent literature and provides data on whether a putative olfactory repellent differentially effects learned and unlearned behavior. We also included a manipulation, also unique in the literature, on whether a putative olfactory repellent has differential effects when the honeybee is allowed to feed or not feed on a sucrose solution. These manipulations, we believe, show the versatility of the proboscis conditioning methodology in the testing of olfactory repellents.

Three hundred twenty honeybees we selected and harnessed from laboratory colonies as described in experiment 1. The bees were divided into 2 main groups consisting of 160 bees. These two main groups were differentiated on the basis of whether butyric acid or DEET was used.

Within each group of 160 bees, 2 subgroups were created based on whether butyric acid (or DEET) was administered when the proboscis was extended to a CS which was earlier trained to elicit a proboscis response (N = 80) or whether it was extended by direct sucrose stimulation to the antennae (N = 80). They were also differentiated based on whether butyric acid (or DEET) was administered while the proboscis was extended and the honeybee permitted to feed (N = 40) or when the proboscis was extended but the honeybee was not permitted to feed (N = 40).

Each honeybee received 4 trials; two trials with butyric acid (or DEET), two with lavender. Lavender was included as a control stimulus to provide an assessment of proboscis retraction to a novel stimulus. If such a control stimulus was not included it would be impossible to determine whether any retraction observed to butyric acid (or DEET) was the result of a repellent effect or the result of novelty. For half of the 40 animals in each subgroup the sequence of presentation was ABBA with A standing for butyric acid (or DEET) and B for lavender. For the remaining 20 honeybees, A stood for lavender and B for butyric acid (or DEET). The duration of stimulus presentation was 3 s, and the intertrial interval 10 min.

The selection process for animals in which the proboscis extension response was elicited by learning required us to condition a sample of bees using a CS of cinnamon and a US of 1.80 M sucrose. Using the procedures outlined in experiment 1 we were able to acquire a sample of bees that always responded to the CS of cinnamon odor.

A trial began by placing a bee in a fume hood, after which the appropriate stimuli were introduced. After application of the stimuli, the animal was returned to a holding area, and a second bee was run. To control for calendar variables bees from all groups were run daily and selected from multiple laboratory hives.

### Statistical methods

Data such as these, in which binomial measurements of the same individual obtained over many trials, are often encountered in research on learning, language, development, and genetics of discrete traits (Lunney 1970; D'Agostino 1971; Katz 1986; Davis 2002; Dupuy et al. 2006; Griswold et al. 2008; Mattila and Smith 2008; Quené and van den Bergh 2008). There are three alternative methods to analyze these types of data: repeated measures ANOVA, non-parametric tests such as Friedman's test, Cochran's Q or its derivatives, and lastly mixed-effects models. Each method has its limitations and the analysis of such data is an active field of statistical research (see Katz 1986, rev. in Davis 2002; Brunner and Puri 2001; Griswold et al. 2008; Baayen et al. 2008).

Experiments 1 and 2 consisted of 12 or 24 repeated measures and experiment 3 used 80 observations per condition. A general linear model for repeated measures analysis of variance was utilized to analyze the data. Winer et al. (1991) citing Cochran (1950), suggest that the probability statements yielded by F-tests are relatively similar to those yielded by equivalent non-parametric tests. Test statistics are reported along with estimates of effect size and observed power. Our use of ANOVA is further justified by our large sample sizes, no missing data, and the measurement of the dependent variable at fixed intervals (see Lunney 1970; Davis 2002).

## Results

### Experiment 1: paired vs. unpaired

**Figure 1.**
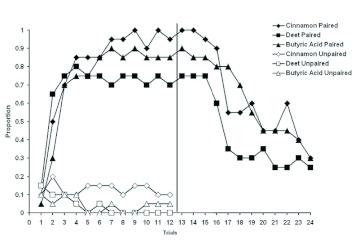
Performance of paired and unpaired *Apis melllifera caucasica* given a conditioned stimulus of either cinnamon, DEET, or butyric acid. The transition from acquisition to extinction occurs on trial 13. Results are reported as the proportion of elicited responses for each trial. High quality figures are available online.

As seen in Figure 1 *A. m. caucasica* rapidly learned to associate the odors of cinnamon, DEET and butyric acid with the sucrose unconditioned stimulus (US). In acquisition training, the proportion of responses to the CS begins low and rapidly increases. After a number of trials, the US was no longer presented with the CS, and extinction took place. Extinction can be seen in the proportion of responses significantly decreasing. The consistently low proportion of bees responding in the unpaired trials indicates that the proportion of responding in the paired group can be attributed to learning. Statistical analysis was used to verify these conclusions.

Three tests were computed to assess the relationships seen in Fig. 1. The first test compared the three paired samples (cinnamon, DEET, and butyric acid). A repeated-measures analysis of variance with trials as the repeated within subjects measure and group (cinnamon, DEET, and butyric acid) as the between subjects variable revealed no significant difference between the three groups (F = 1.81; df = 2,57; P = 0.17, partial η^2^ = 0.06; power = 0.36).

The second test was a comparison of paired vs. unpaired groups using a repeated measures analysis of variance with trial as the repeated within subjects measure and group as the between subjects variable. The repeated measures ANOVA revealed a highly significant difference between paired and unpaired groups (F = 56.93; df = 5,114; P < 0.0001; partial η^2^ = 0.71; power = 1.00). The differences between paired and unpaired performance indicated that the increase in the proportion of animals responding to the three different CSs was the result of a learned association.

The last test conducted was a comparison of paired vs. extinction. A paired-samples t-test was utilized to compare the proportion means of acquisition and extinction. Results indicate a significant difference between acquisition (M = 0.83, SD = 0.38) and extinction (M = 0.28, SD = 0.45), t (59) = 7.97; P < 0.0001; power = 1.00.

### Experiment 2: Diluted butyric or DEET as unconditioned stimuli, paired vs. unpaired comparisons.

The acquisition curves when cinnamon odor was paired with sucrose were not significantly different from those shown in Figure 1. In contrast, when cinnamon odor was either paired with diluted butyric acid or DEET, the curves resembled the unpaired curves of Figure 1. No conditioning was evident. When diluted butyric acid was used only 2 CS responses out of a possible 240 were observed (20 subjects × 12 CS presentations) in honeybees receiving paired CS-US presentations with no CS responses observed after the 2^nd^ CS presentation. Honeybees in the unpaired group responded only 3 times out of 240 opportunities to the CS with none after the 2^nd^ CS presentation. The data were similar when diluted DEET was used as a US. Of 240 possible CS responses in honeybees receiving paired CS-US presentations only 8 were observed with none after the 6^th^ training trial. The number of CS responses in the unpaired group showed a similar pattern with 17 CS responses observed out of 240 opportunities with no responses observed after the 7^th^ CS presentation.

The failure to find paired vs. unpaired differences when diluted butyric acid or diluted DEET was confirmed by statistical analysis. A repeated-measures analysis of variance with trials as the repeated within subjects measure and group (DEET paired vs. DEET unpaired) as the between subjects variable revealed no significant difference between the two groups (F = 0.12; df = 1,38; P = 0.73; partial η^2^ = 0.003; power = 0.06). A second repeated-measures ANOVA was run for butyric acid. Trials was the repeated within subjects measure and group (butyric acid paired vs. butyric acid unpaired) was the between subjects variable. This test also revealed no significant difference between the paired and unpaired group (F = 0.22; df = 1,38; P = 0.64; partial η^2^ = 0.01; power = 0.07). The results show that neither DEET nor butyric acid diluted with sucrose functioned as a US to support conditioning.

**Figure 2.**
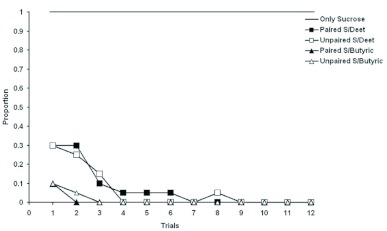
Comparison of unconditioned responses of *Apis melllifera caucasica* to sucrose alone, sucrose mixed with DEET, or sucrose mixed with butyric acid in animals receiving paired or unpaired training. Results are reported as the proportion of elicited responses for each trial. High quality figures are available online.

Figure 2 shows that the reason why there was no conditioning to the cinnamon CS when paired with a US of either diluted butyric acid or diluted DEET was that such a US seldom elicits an unconditioned feeding response. Some animals fed on the diluted butyric acid or diluted DEET the first time they awerere presented but as training continues the response to the US rapidly declined. In contrast, when untreated sucrose was used as the unconditioned stimulus all honeybees responded on all trials.

### Experiment 3: Conditioned suppression

**Figure 3.**
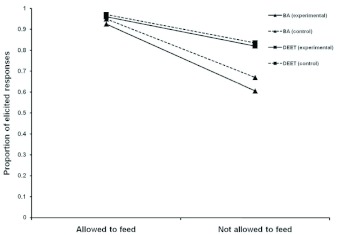
Effect of butyric acid (BA), DEET (25%), and lavender odor (control odor) on proboscis extension by *Apis melllifera caucasica* elicited by antenna stimulation. Results are reported as the proportion of elicited responses. High quality figures are available online.

Figure 3 shows that conditioned suppression resulted when proboscis extension was elicited by antennae stimulation with sucrose (i.e. the unlearned conditioned) and were permitted to feed or prevented from feeding. When the proboscis was extended by sucrose stimulation, and animals were subsequently permitted to feed on sucrose, few honeybees retracted their proboscis when the odor of butyric acid, DEET, or lavender was applied. In contrast, when the odor of butyric acid, DEET, or lavender was applied to the extended proboscis, and honeybees were not permitted to feed, more honeybees retracted their proboscis to the odor of butyric acid, DEET, or lavender. That honeybees also retracted their proboscis to lavender odor when not permitted to feed provides further evidence that the putative olfactory repellents may be effective in part because of stimulus novelty effects. The odors of DEET and butyric acid were no more effective in retracting the extended proboscis than the control odor. It is important to note that the odor of lavender was used to control for the effect of novelty per se. Without such a control any retraction of the proboscis in response to the test odors could have been interpreted as simply a novelty effect.

Statistical analysis of the data presented in Figure 3 is consistent with this interpretation and indicates that when butyric acid was applied to an extended proboscis, the proboscis did not retract compared to a control odor of lavender (F = 0.45; df = 1,78; P = 0.50; partial η^2^ = 0.006). The effect of applying butyric acid was negligible. There were no significant differences between groups. That is, the number of proboscis extensions elicited by learning or sucrose stimulation did not differ (F = 0.25; df = 1,158; P = 0.62; partial η^2^ = 0.002). The same results were achieved using DEET. When DEET was applied to an extended proboscis, the proboscis did not retract compared to the control odor of lavender (F = 0.11; df = 1,78; P = 0.74; partial η^2^ = 0.001). The effect of applying DEET was also negligible. Again, no significant difference between groups in which the proboscis extension was elicited by learning or by sucrose stimulation was found (F = 2.84; df = 1,158; P = 0.09; partial η^2^ = 0.02).

**Figure 4.**
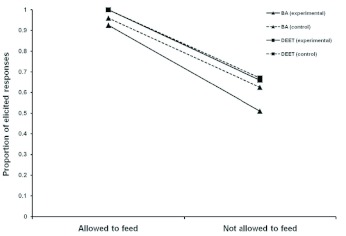
Effect of butyric acid (BA), DEET (25%), and lavender (control odor) on proboscis extension elicited by learning in *Apis melllifera caucasica*. Results are reported as the proportion of elicited responses. High quality figures are available online.

Figure 4 shows that conditioned suppression resulted when proboscis extension was elicited by cinnamon odor which was previously associated with sucrose (i.e. the learned conditioned). The results were similar to those shown in Figure 3. When proboscis extension was elicited by a conditioned stimulus odor and honeybees were allowed to feed, the odors of butyric acid, DEET, and lavender had little effect on the extended proboscis. In contrast, honeybees retraced their proboscis when exposed to these odors and were not permitted to feed.

The same statistical analyses that were run on Figure 3 were run on Figure 4. An ANOVA revealed a significant difference between the allowed to feed and not allowed to feed groups in Figure 4 (F = 156.40; df = 1,328; P < .001; partial η^2^ = .330) thereby replicating the results in Figure 3.

The difference in proboscis retraction between the allowed to feed and not allowed across learned and unlearned subgroups for butyric acid was analyzed. There was a significant difference (F = 59.03; df = 1,158; P < 0.001; power = 1.00). This result suggests that if allowed to feed, honeybees will continue proboscis extension, even in the presence of butyric acid. If the honeybees were not allowed to feed, the proboscis was retracted. This trend was also seen in the lavender control group (F = 50.25; df = 1,158; P < 0.001; power = 1.00). The same results were found for the DEET and lavender controls. DEET (F = 34.89; df = 1,158; P < 0.001; power = 1.00), lavender (F = 35.64; df = 1,158; P < 0.001; power = 1.00).

An analysis was also conducted comparing proboscis extension elicited by learning or by sucrose stimulation collapsed across groups. No significant difference was found (P > .11). This suggests that the effect our test odors on an extended proboscis was independent of how it was elicited.

## Discussion

These results demonstrate that the odor of DEET or butyric acid is not intrinsically an olfactory repellent to honeybees since the odor can serve as a cue signaling food. However, when mixed with high molarity sucrose, these compounds will not support classical conditioning of the proboscis extension. This is consistent with the idea that these chemicals represent gustatory repellents when in direct contact, but not as an olfactory repellent. Honeybees will consume little if any of the test solutions. This result is in agreement with a recent study on DEET (Ditzen et al. 2008, but see Syed and Leal 2008) and another study using both the proboscis conditioning paradigm and free-flying situations to show that honeybees will not consume a pesticide made from essential oils but can use these odors to signal a food source (Abramson et al. 2006a).

When a conditioned suppression paradigm was used the results again supported the notion that DEET and butyric acid are not olfactory repellents for honeybees. They do not lead to greater suppression of proboscis extension than a novel stimulus (lavender). However, there were feeding related differences when odor was applied to a bee with an extended proboscis. More animals kept their proboscis extended when the odors were applied during feeding than when the odors were applied and honeybees not allowed to feed. This result suggests that if allowed to feed, honeybees will continue proboscis extension, even in the presence of novel odors. If the honeybees are not allowed to feed, the proboscis is retracted.

Repellents are stimuli that interfere with reproduction, foraging, and feeding (Apfelbach et al. 2005; Raguso 2008). Repellents, such as pheromones or kairomones, could affect the behavior of organisms across the range of the animal kingdom. Pheromones occur in contexts extending from oviposition (Averill and Prokopy 1987; Ganesan et al. 2006) to mate choice where males may reduce the probability of second mating by marking mated females (Seidelmann et al. 2003). Kairomones are especially important in interactions across taxa in contexts such as prey-predator interactions (Apfelbach et al. 2005) or plant-pollinator interactions, and may act as filters against non-specialized visitors or modify behavior of legitimate visitors (Kessel and Baldwin 2006; Agarwal and Rastogi 2008; rev. in Raguso 2008).

Repellents also aim to interfere with feeding and distribution of target organisms (Baker et al. 2005; Isman 2006; Werner et al. 2007; Carroll et al. 2008). However, the evidence for repellent action is often just the modification in some targeted behavior (Xue et al. 2001). This evidence may lead to misidentification of mechanisms of action. In particular, it may lead to the mistaken interpretation that the change in the insect's behavior is the result of a repellent but in reality it is due to stimulus novelty. If this is indeed the case, the response to novelty may later lead to attraction once novelty is reduced. This is similar to recent reports that the mechanism of DEET action is through odor masking (with evidence that a familiar attractant resulted in less responses in the presence of DEET, Ditzen et al. 2008) or through avoidance (with evidence that DEET is perceived and avoided, Syed and Leal 2008). The effect of butyric acid in practical use has been suggested to be an olfactory repellent. In fact, in the current study and both recent studies (Ditzen et al. 2008 and Syed and Leal 2008), direct contact with undiluted putative repellents (direct contact or mixed in food) led to inhibitory responses. In our study, these chemicals were not repellent as odors (similar to inference by Ditzen et al. 2008 for DEET).

We believe the use of conditioning paradigms such as proboscis conditioning has much to recommend for the study of olfactory and gustatory repellents. The experiments are easy to perform and much data can be obtained not only on learned behavior to a conditioned stimulus, but on reflexive behavior as represented by the unconditioned response as well. In addition, it is well known that a specialized part of insect brain, the mushroom bodies, are critical for conditional learning tasks and behavioral development in flies and bees (Davis 1993; Erber et al. 1980; Heisenberg 1980; Heisenberg et al. 1985; Withers et al. 1993; Fahrbach et al. 1995, 1997; Menzel and Giurfa 2001; Giurfa 2007). There is anatomical evidence that tactile, visual, gustatory, and olfactory information reaches the mushroom bodies (e.g. Critedden et al. 1998; Schröter and Menzel 2003). The separate responses to different modalities for the same stimulus (chemical) could be important in understanding how different types of information are processed in the insect brain.

We do not advocate replacing field and neurophysiological tests with proboscis conditioning assays but suggest that the strongest evidence for a putative olfactory repellent are successful field and neurophysiolological tests coupled with the failure to find proboscis conditioning to a CS consisting of the putative olfactory repellent.

## **Abbreviations**

CS: conditioned stimuli;
US: unconditioned stimuli

## References

[bibr01] Abramson CI (1994). *A primer of invertebrate learning: the behavioral perspective.*.

[bibr02] Abramson CI (1986). Aversive conditioning in honeybees (*Apis mellifera*).. *Journal of Comparative Psychology*.

[bibr03] Abramson CI, Bitterman ME (1986a). Latent inhibition in honeybees.. *Animal Learning and Behavior*.

[bibr04] Abramson CI, Bitterman ME (1986b). The US-preexposure effect in honeybees.. *Animal Learning and Behavior*.

[bibr05] Abramson CI, Boyd BJ (2001). An automated apparatus for conditioning proboscis extension in honeybees (*Apis mellifera* L.).. *Journal of Entomological Science*.

[bibr06] Abramson CI, Aquino SI, Azeredo GA, Filho JR, Price JM (1997). The attraction of africanized honeybees (*Apis mellifera* L.) to soft drinks and perfumes.. *Journal of General Psychology*.

[bibr07] Abramson CI, Nolf SL, Mixson TA, Wells H (2010). Can honey bees learn the removal of a stimulus as a conditioning cue?. *Ethology,* in press.

[bibr08] Abramson CI, Singleton JB, Wilson MK, Wanderley PA, Ramalho FS, Michaluk LM (2006a). The effect of an organic pesticide on mortality and learning in africanized honeybees (*Apis mellifera* L.) in Brasil.. *American Journal of Environmental Sciences*.

[bibr09] Abramson CI, Wilson MK, Singleton JB, Wanderley PA, Wanderley MJ, Michaluk LM (2006b). Citronella is not a repellent to Africanized honeybees *Apis mellifera* L. (Hymenoptera: Apidae).. *BioAssay*.

[bibr10] Agarwal VM, Rastogi N (2008). Role of floral repellents in the regulation of flower visits of extrafloral nectar-visiting ants in an Indian crop plant.. *Ecological Entomology*.

[bibr11] Apfelbach R, Blanchard CD, Blanchard RJ, Hayes RA, McGregor IS (2005). The effects of predator odors in mammalian prey species: a review of field and laboratory studies.. *Neuroscience and Biobehavioral Reviews*.

[bibr12] Atkins Jr. EL, McDonald RL, Greywood-Hale EA (1975a). Repellent additives to reduce pesticide hazards to honeybees: field tests.. *Journal of Environmental Entomology*.

[bibr13] Atkins EL, McDonald RL, McGouern TD, Beroza M., Greywood-Hale E (1975b). Repellent additives to reduce pesticide hazards to honeybees: laboratory testing.. *Journal of Apiculture Research*.

[bibr14] Averill AL, Prokopy RJ (1987). Residual activity of oviposition-detering pheremone in *Rhagoletis pomonella* (Diptera: Tephritidae) and female response to infested fruit.. *Journal of Chemical Ecolology*.

[bibr15] Baayen RH, Davidson DJ, Bates DM (2008). Mixed-effects modeling with crossed random effects for subjects and items.. *Journal of Memory and Language*.

[bibr16] Baker SE, Elwood SA, Watkins R, MacDonald DW (2005). Non-lethal control of wildlife: using chemical repellents as feeding deterrents for the European badger Meles meles.. *Journal of Applied Ecolology*.

[bibr17] Blum MS, Fales HM, Tucker KW, Collins AM (1978). Chemistry of the sting apparatus of the worker honeybee.. *Journal of Apiculture Research*.

[bibr18] Bodur C, Kence M, Kence A (2007). Genetic structure of honeybee, *Apis mellifera* L. (Hymenoptera: Apidae) populations of Turkey inferred from microsatellite analysis.. *Journal of Apicultre Research*.

[bibr19] Brunner E, Puri ML (2001). Non-parametric methods in factorial designs.. *Statistical Papers*.

[bibr20] Carroll JF, Benante JP, Klun JA, White CE, Debboun M, Pound JM., et al (2008). Twelve-hour duration testing of cream formulations of three repellents against *Ambylomma americanum.*. *Medical and Veterinary Entomology*.

[bibr21] Cochran WG (1950). The comparison of percentages in matched samples.. *Biometrika*.

[bibr22] Crittenden JR., Skoulakis EMC, Han KA., Kalderon D., Davis R.L (1998). Tripartice mushroom body architecture revealed by antigenic markers.. *Learning and Memory*.

[bibr23] D'Agostino R (1971). A second look at analysis of variance on dichotomous data.. *Journal of Educational Measurement*.

[bibr24] Davis RL (1993). Mushroom bodies and *Drosophila* learning.. *Neuron*.

[bibr25] Davis CS (2002). *Statistical methods for analysis of repeated measurements.*.

[bibr26] Ditzen M, Pellegrino M, Vosshall LB (2008). Insect odorant receptors are molecular targets of the insect repellent DEET.. *Science*.

[bibr27] Dupuy F, Sandoz J-C, Giurfa M, Josens R (2006). Individual olfactory learning in *Camponotus* ants.. *Animal Behavior*.

[bibr28] Erber J, Masuhr T, Menzel R (1980). Localization of short-term memory in the brain of the bee, *Apis mellifera.*. *Physiological Entomology*.

[bibr29] Estes WK, Skinner BF (1941). Some quantitative properties of anxiety.. *Journal of Experimental Psychology*.

[bibr30] Fahrbach SE, Robinson GE (1995). Behavioral development in the honeybee: toward the study of learning under natural conditions.. *Learning and Memory*.

[bibr31] Fahrbach SE, Giray T, Robinson GE (1995). Volume changes in the mushroom bodies of adult honeybee queens.. *Neurobiology of Learning and Memory*.

[bibr32] Fahrbach SE, Farris SM, Robinson GE, Giray, T (1997). Expansion of the neuropil of the mushroom bodies in male honeybees is coincident with initiation of flight.. *Neuroscience Letters*.

[bibr33] Free JB (1987). *Pheremones of social bees.*.

[bibr34] Free JB, Ferguson AW, Simpkins JR (1989). Honeybee responses to chemical components from the worker sting apparatus mandibular glands in field tests.. *Journal of Apiculture Research*.

[bibr35] Ganesan K, Mendki MJ, Suryanarayana MV, Prakash S, Malhotra RC (2006). Studies of *Aedes aegypti* (Diptera: Culicidae) ovipositional reponses ot newly identified semiochemcials from conspecific eggs.. *Australian Journal of Entomology*.

[bibr36] Giurfa M (2007). Behavioral and neural analysis of associative learning in the honeybee: a taste from the magic well.. *Journal of Comparative Physiology A*.

[bibr37] Graham JM (1992). *The hive and the honeybee.*.

[bibr38] Griswold CK, Gomulkiewicz R, Heckman N (2008). Hypothesis testing in experimental studies of function-valued traits.. *Evolution*.

[bibr39] Heffernan DJ, Andelt WF, Shivik JA (2007). Coyote investigative behavior following removal of novel stimuli.. *Journal of Wildlife Management*.

[bibr40] Heisenberg M, Siddigui O, Babu P, Hall LM, Hall JC (1980). Mutants of brain structure and function: what is the significance of the mushroom bodies for behaviour?.

[bibr41] Heisenberg M, Borst A, Wagner S, Byers D (1985). *Drosophila* mushroom body mutants are deficient in olfactory learning.. *Journal of Neurogenetics*.

[bibr42] Isaac B, Hoffman CM (2002). *n-Butyric anhydride* — *abiotic biodegradation: hydrolysis.* Final Report #1204-HYD..

[bibr43] Isman MB (2006). Botanical insecticides, deterrents, and repellents in modern agriculture in an increasingly regulated world.. *Annual Review of Entomology*.

[bibr44] Kandemir I, Kence M, Kence A (2000). Genetic and morphometirc variation in honeybee (*Apis mellifera*) populations of Turkey.. *Apidologie*.

[bibr45] Katz BM (1986). Analyzing dichotomous data from two correlated samples measured at two points in time.. *Journal of Experimental Education*.

[bibr46] Kessler D, Baldwin IT (2006). Making sense of nectar scents: the effects of nectar secondary metabolites on floral visitors of *Nicotiana attenuata.*. *The Plant Journal*.

[bibr47] Lunney G (1970). Using analysis of variance with a dichotomous dependent variable: an empirical study.. *Journal of Educational Measurement*.

[bibr48] Malerbo-Souza DT, Nogueira-Couto RH (2004). Efficiency of n-Octyl-Acetate, 2-Heptanone and citronella in repelleing bees from Brasil (Ocimum sellowii - Labiatae).. *Brazilian Archives of Bioliology and Technology*.

[bibr49] Masetti A, Maini S (2006). Arm in cage tests to compare skin repellents against bites of *Aedes albopictus.*. *Bulletin of Insectology*.

[bibr50] Mattila HR, Smith BH (2008). Learning and memory in workers reared by nutritinonally stressed honeybee (*Apis mellifera* L.) colonies.. *Physiology and Behavior*.

[bibr51] Menzel R, Giurfa M (2001). Cognitive architecture of a mini-brain: the honeybee.. *Trends in Cognitive Sciences*.

[bibr52] Quené H, van den Bergh H (2008). Examples of mixed-effects modeling with cross random effects and with biomial data.. *Journal of Memory and Language*.

[bibr53] Page RE Jr., Erber J, Fondrk MK (1998). The effect of genotype on response thresholds to sucrose and foraging behavior of honeybees (*Apis mellifera* L.).. *Journal of Comparative Physiology A*.

[bibr54] Raguso RA (2008). Start making scents: the challenge of integrating chemistry into pollination ecology.. *Entomologia Experimentalis et Applicata*.

[bibr55] Schreck CE (1977). Techniques for the evaluation of insect repellents: a critical review.. *Annual Review of Entomology*.

[bibr56] Schröter U, Menzel R (2003). A new ascending sensory tract to the calyces of the honeybee mushroom body, the subesophageal-calycal tract.. *Journal of Comparative Neurology*.

[bibr57] Seidelmann K, Weiner H, Ferenz HJ (2003). Wings and legs are productions sites for the desert locust courtship-inhibition pheremone, phenylacetonitrile.. *Journal of Insect Physiology*.

[bibr58] Smith BH, Abramson CI, Tobin TR (1991). Conditioned withholding of proboscis extension in honeybees (*Apis mellifera*) during discriminative punishment.. *Journal of Comparative Psychology*.

[bibr59] Syed Z, Leal WS (2008). Mosquitoes smell and avoid the insect repellent DEET.. *Proceedings of the National Academy of Sciences, USA*.

[bibr60] Werner SJ, Cummings JL, Tupper SK, Stahl RS, Primus TM, Hurley, JC (2007). Caffeine formulation for avian repellency.. *Journal of Wildlife Management*.

[bibr61] Winer BJ, Brown DR, Michels KM (1991). *Statistical principles in experimental design*.

[bibr62] Withers GS, Fahrbach SE, Robinson GE (1993). Selective neuroanatomical plasticity and division of labour in the honeybee.. *Nature*.

[bibr63] Xue RD, Barnard DR, Ali A (2001). Laboratory and field evaluation of insect repellents as oviposition deterrents against the mosquito *Aedes albopictus.*. *Medical and Veterinary Entomology*.

[bibr64] Zhang S, Bock F, Si A, Tautz J, Srinivasan MV (2005). Visual working memory in decision making by honeybees.. *Proceedings of the National Academy of Sciences, USA*.

